# Manuka Honey Inhibits Biofilm Formation and Reduces the Expression of the Associated Genes in *Pectobacterium brasiliense*

**DOI:** 10.1155/2024/8837149

**Published:** 2024-10-28

**Authors:** Tri Joko, Sheila Ava, Isna Nurifa Sasmita Putri, Siti Subandiyah, Muhammad Saifur Rohman, Naoto Ogawa

**Affiliations:** ^1^Department of Plant Protection, Faculty of Agriculture, Universitas Gadjah Mada, Yogyakarta 55281, Indonesia; ^2^Department of Microbiology, Faculty of Agriculture, Universitas Gadjah Mada, Yogyakarta 55281, Indonesia; ^3^Department of Applied Life Sciences, Faculty of Agriculture, Shizuoka University, Shizuoka 422-8529, Japan

## Abstract

Biofilms are major virulence factors formed by pathogenic bacteria to invade their host and maintain their colony. While biofilms usually develop on diverse solid surfaces, floating biofilms, also called pellicles, are formed at the air–liquid interface. To address the problem of biofilm formation by bacterial pathogens, honey has been extensively studied. However, information on the effect of honey on biofilm formation by plant pathogens is scarce. This study aimed to determine the effects of manuka honey on biofilm and pellicle formation by *Pectobacterium brasiliense* and analyze the expression of genes encoding proteins needed to form biofilm by using semiquantitative PCR and RT-qPCR. Treatment with 5% (w/v) of manuka honey significantly decreased biofilm and pellicle formation by *P. brasiliense*. RT-qPCR results showed that the expression of *bcsA*, *fis*, *hrpL*, and *expI* decreased 7.07-fold, 5.71-fold, 13.11-fold, and 6.26-fold, respectively, after exposure to 5% (w/v) manuka honey. Our findings reveal that manuka honey may effectively inhibit biofilm and pellicle formation.

## 1. Introduction

A biofilm is a three-dimensional accumulation of bacterial communities that adheres to solid surfaces and is surrounded by a self-produced matrix of extracellular polymeric substances (EPSs) [1]. EPS is mainly composed of polysaccharides, proteins, nucleic acids, and lipids, all of which maintain the stability of the film and facilitate its cohesion and surface adhesion [2]. The combination of these biomolecules is called a matrixome and influences bacterial virulence [3]. Biofilms are an important component of bacterial functions. Bacterial transition from motility to biofilm formation happens when their motility is inhibited due to an increase in the levels of c-di-GMP, which in turn, slows down the bacteria and inhibits the expression of the flagellar genes [4]. This transition between the two states is an example of bacterial adaptation to environmental signals and stress [5]. Some Gram-negative bacteria, including pathogenic bacteria, can form a pellicle, which is a biofilm that floats on the air–liquid interface under static conditions [6]. Biofilms, then, contribute significantly to pathogenicity. Some pathogenic microorganisms can only regulate and express their virulence if environmental conditions and cell density are optimal. The production of biofilms is one of the most intriguing tactics to increase fitness against the harsh environments on or in the plant. Microbial biofilms can grow on the surfaces of leaves, roots, and plant tissues' intercellular spaces.

Plant-associated bacteria interact with the surface of plant tissues prior to pathogenesis process through symbiotic relationships and commensalism [7]. The surface components of plant tissues, the availability of nutrients and water, and the nature of bacterial colonies affect the formation of biofilm structures [8, 9]. Certain secondary plant metabolites, including alkaloids, flavonoids, phenols, glycosides, steroids, saponins, and terpenoids, have been shown to hinder biofilm formation by preventing and interrupting the mature biofilm and the bacterial cells' ability to disperse, mainly damaging the cell membranes [10, 11]. However, it should be noted that these plant-derived compounds are not directly released from the host plant into bacterial biofilm [12]. In addition, Guttman et al. [13] reported that the leaf texture with larger roughness of the abaxial leaf of *Zantedeschia aethiopica* is more resistant to *Pectobacterium* spp. than members of the other Araceae*. Pectobacterium* species can recognize hosts and express various virulence factors, and then plant hosts activate defense mechanisms in response to the identified bacterial strain [14]. Endophytic and pathogenic bacteria living in vascular tissues are known to be capable of invading plant tissues and forming biofilms. Inside the biofilm, phytopathogenic bacteria have higher virulence and pathogenicity that stand up to antimicrobials synthesized by the host plant and thus can act coordinated to colonize host plants and eventually infect them. All these processes are far more challenging for bacteria attempting them individually. One advantage of biofilm formation is that it shields connected bacteria from environmental stressors like UV radiation and desiccation [15, 16]. The formation of biofilms can cause blockage and tissue damage in xylem cells [17]. The ability to form robust biofilms inside xylem vessels in *Xylella fastidiosa* is the main cause of diseases, such as Pierce's disease and citrus variegated chlorosis; however, in certain instances, biofilms aid in limiting rapid growth and reducing self-virulence. This allows the pathogen to survive as a commensal endophyte in many plant species rather than instantly destroying the plant host [18]. *Pectobacterium brasiliense*, an phytopathogenic bacterium that is highly concentrated in the xylem, produces large amounts of plant cell-wall degrading enzymes that can cause necrosis of the vascular tissues [19, 20]. This pathogen has been widely reported to cause soft rot disease in potatoes, ornamental crops, and other economically valuable crops [21–24]. Biofilm formation by *P. brasiliense* plays an important role in bacterial colonization and disease progression [25]. *P. brasiliense* in susceptible potato cultivars colonizes xylem tissue to form biofilm-like aggregates that could lead to occlusion problems in some tissues [26, 27].

Honey has been extensively studied as a natural compound that could address the propensity of bacteria to form biofilms [28–30]. Manuka is a type of honey that has been widely investigated for its ability to inhibit the biofilm formation of various pathogenic bacteria. However, the effect of manuka honey on the biofilm formation of plant pathogens has not been extensively studied. Thus, the purpose of the present study is to determine the effects of manuka honey on the biofilm and pellicle formation of *P. brasiliense* and the expression of the related genes.

## 2. Materials and Methods

### 2.1. Bacterial Isolate and Growth Condition

*P. brasiliense* Pal3.4 used in this study was grown on yeast peptone agar medium (YPA; 0.5% yeast extract, 1% polypeptone, 1.5% agar). A single bacterial colony was incubated for 1-2 days at room temperature (±27°C) on a new YPA medium to maintain the isolate's viability and purity. Colonies of *P. brasiliense* were scraped from forty-eight-hour-old isolates and suspended in sterile water to an OD600 nm of 0.2 (108 CFU/mL) measured with a spectrophotometer (Genesys 10S UV-VIS, Thermo Fisher Scientific, USA). A 50 *μ*L of bacterial suspension was then grown in 5 mL of yeast peptone broth (YPB) medium. Manuka honey (Streamland UMF 20+) was added to the medium at a concentration of 5% (w/v) as previously described [31]. A control treatment without adding manuka honey (0% w/v) to the medium was also prepared.

### 2.2. Assessment of Biofilm Formation by Crystal Violet Staining Assay

The biofilm formation protocol was adapted from the work of O'Toole and Kolter [32] with slight modifications. Biofilm formation was assayed in terms of the ability of cells to adhere to a bottle made of polyvinyl chloride (PVC). An overnight culture grown in SOBG medium (20 g/L tryptone, 5 g/L yeast, 0.5 g/L NaCl, 2.4 g/L MgSO_4_, 0.18 g/L KCl, 40% glycerol) was diluted (1:10 v/v) with the same medium containing 5% (w/v), 10% (w/v), 25% (w/v), 50% (w/v), and 75% (w/v) concentration of manuka honey as a treatment. The honey was replaced with sterile distilled water in the control treatment. The cultures (5 mL, approximately 10^5^ CFU) were poured into PVC bottles and incubated for 48 h at room temperature without shaking. The cultures were removed after incubation, and the absorbance was measured with a spectrophotometer (Genesys 10S UV-VIS, Thermo Fisher Scientific, USA) in OD_600nm_. The bottle was dried for 15 min, rinsed thrice with sterile distilled water, added with 7.5 mL of 1% crystal violet solution, and allowed to stand for 20 min. The dye stained the cells but not the plastic surface. The bottles were washed three times with sterile distilled water until no color was observed in the rinse water. The final wash was done with 7.5 mL of 96% ethanol solution and left for 2 min. 3 mL of this ethanol solution was measured with a spectrophotometer at the absorbance of 600 nm using a Genesys 10S UV-VIS (Thermo Fisher Scientific, USA).

### 2.3. Assessment of Pellicle Formation

The pellicle formation assay was adapted from the previously described protocol [33]. In brief, overnight bacterial cultures were diluted at a ratio of 1:100 (v/v) in 5 mL of SOBG medium containing 5% manuka honey (w/v) as treatment and without manuka honey as control and grown in the dark in glass tubes for 72 h at room temperature without shaking. Finally, the appearance of pellicles on water surface of treatment and control was recorded.

### 2.4. Selection and Primer Design of Genes Involved in Biofilm and Pellicle Formation

PCR primers were designed to amplify genes involved in biofilm and pellicle formation by *P. brasiliense*, including *bcsA* (cellulose synthase), *fis* (DNA-binding protein), *hrpL* (alternative sigma factor), and *expI* (quorum-sensing (QS) signal generator, acyl-homoserine lactone (AHL)) [34–37]. The *recA* (Recombinase A) gene, a known housekeeping gene in *P. brasiliense*, was used as an internal control. The primer was designed on the basis of the complete genome sequence of *P. brasiliense* type strain LMG 21371^T^ (accession number GCA_000754695.1), which is available at the National Center for Biotechnology Information database (https://www.ncbi.nlm.nih.gov/). The selected primer candidates were searched using BLAST (https://blast.ncbi.nlm.nih.gov/Blast.cgi) to confirm the identity of the gene to be used. The primers were designed using Primer3Plus software (https://primer3plus.com/cgi-bin/dev/primer3plus.cgi), and the annealing temperature of each primer was optimized using a gradient thermal cycler [38]. The primer sequences are shown in Table 1.

### 2.5. Bacterial RNA Isolation and cDNA Synthesis

*P. brasiliense* was grown in YPB medium for 12 h with or without the addition of 5% manuka honey. The bacterium was harvested by centrifugation of 100,00 × g for 1 min, and RNA was isolated using GENEzol Reagent (Geneaid, Taiwan) according to the manufacturer's instructions. The quality and quantity of RNA were calculated using a MaestroNano spectrophotometer (MaestroGen, Taiwan) [39].

### 2.6. Analysis of Biofilm and Pellicle Gene Expression

Bacterial RNA was synthesized into cDNA via the PCR reverse-transcription method using ReverTra Ace-*α*-® (TOYOBO, Japan) with the primer sequences presented in Table 1. In this study, gene expression analysis was carried out using two methods, namely, semiquantitative gene expression levels and quantitative gene relative expression. Semiquantitative analysis of the expression of genes encoding for biofilm and pellicle formation was performed using conventional PCR (Bio-Rad T100, Germany) with GoTaq Green Master Mix (Promega, USA). PCR was performed as follows: initial denaturation at 95°C for 3 min, followed by 30 cycles of denaturation at 95°C for 1 min, annealing (Table 1) for 40 s, and extension at 72°C for 40 s. Visualization was performed by electrophoresis and UV transillumination (Bio-Rad, USA) [40]. ImageJ software [41] was used to quantify gel images of biofilm and pellicle gene expression in comparison with that of *recA*. Gene expression was clustered into biofilm and pellicle formation–associated genes with those of *recA*. Expression levels were quantified from gel images from pellicle and biofilm-related genes compared to its reference gene. Quantitative analysis was performed using a CFX96 Touch real-time qPCR (Bio-Rad, USA) with THUNDERBIRD SYBR qPCR Mix (TOYOBO, Japan) and the *recA* gene as an internal standard. The program used included initial denaturation at 95°C for 1 min, followed by 39 cycles of denaturation at 95°C for 15 s, annealing at 59°C for 30 s, and extension at 72°C for 5 s and final extension at 72°C for 5 min. Results were analyzed as described by Livak and Schmittgen [42] comparing the CT of the target gene with the CT of the reference gene. ΔCT = Ct target gene − Ct internal control, ΔΔCT = ΔCT treatment − ΔCT control, and relative expression = 2^−ΔΔCT^. The fold change differences between 0% and 5% treatments were calculated by dividing the expression fold change of 0% treatment by the expression fold change of 5% treatment for each biofilm and pellicle-related genes.

## 3. Results

### 3.1. Reduction in Biofilm Formation After Honey Treatment

**Figure 1:**
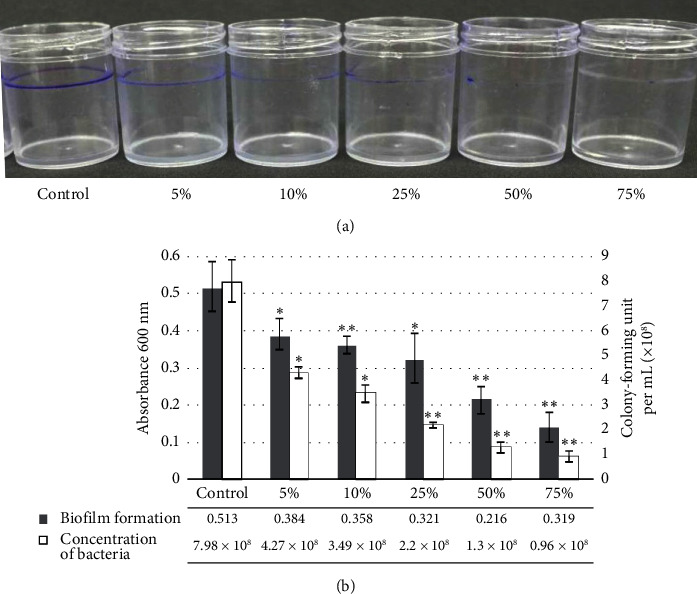
Assay of biofilm formation by *Pectobacterium brasiliense* by crystal violet staining. Treatments: 5%, 10%, 25%, 50%, and 75% honey; control, no honey. Biofilm formation was characterized as a purple ring appearing around the sides of the polyvinyl chloride bottles (a) as well as by solubilizing the dye in ethanol and determining the absorbance at 600 nm; bacterial concentration was also measured (b). Three independent experiments gave similar results. ⁣^∗^(*p* < 0.05) and ⁣^∗∗^(*p* < 0.01) are significantly different from the control as evaluated using *t*-test.

The biofilm formation of *P. brasiliense* in the SOBG medium was observed as a purple ring on the top of the PVC surface. After staining with CV, treatment of 5% manuka honey reduced biofilm formation, which was adhered to the PVC surface, followed by 10%, 25%, 50%, and 75%, while the control was not (Figure 1(a)). Biofilm formation measurement showed that all treatments decreased in absorbance, and the concentration of bacteria was also reduced (Figure 1(b)). Absorbance measurement showed that 7.98 × 10^8^ CFU/mL of control can form biofilm formation adhered to PVC surface at 0.513 of OD600nm, and treatment with 5% manuka honey can reduce the bacteria to 4.27 × 108 CFU/mL and biofilm formation decreased to 0.384. In addition, treatment with 10%, 25%, 50%, and 75% showed similar reduction results (Figure 1(b)). This result showed that the control treatment did not show any inhibition of biofilm formation compared to the manuka honey treatment.

### 3.2. Pellicle Formation

**Figure 2:**
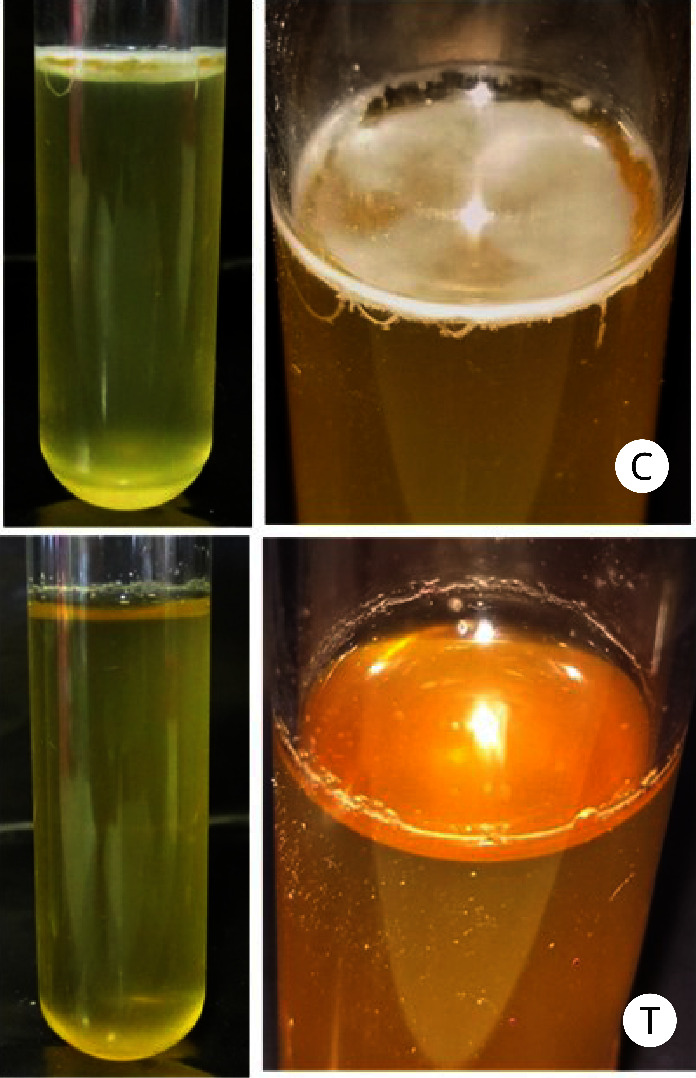
Pellicle formation by *Pectobacterium brasiliense* grown in SOBG medium and analyzed after 72 h of incubation at room temperature in the absence of light. A pellicle developed at the liquid–air interface in control (without manuka honey) treatment (C). No pellicle formation was observed in the culture treated with 5% manuka honey (T). Three independent experiments gave similar results.

A pellicle is a robust layer of connected cells covering the surface of a liquid. After 72 h of incubation in test tubes, *P. brasiliense* demonstrated pellicle formation, as evidenced by the formation of a thick aggregate of cells on the surface of the liquid SOBG culture medium without manuka honey treatment. Compared with the control treatment, reductions in pellicle formation were observed on the surface of the liquid medium treated with 5% manuka honey (Figure 2). These results indicated that manuka honey inhibits the pellicle formation of *P. brasiliense*.

### 3.3. Semiquantitative Analysis of Gene Expression

**Figure 3:**
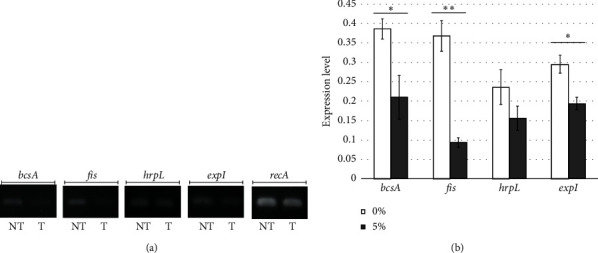
Semiquantitative analysis of the expression of genes involved in biofilm and pellicle formation in *Pectobacterium brasiliense*. (a) Visualization of electrophoresed gels without (NT) and with (T) 5% manuka honey treatment. (b) Estimation of gene expression level without (0%) and with manuka honey treatment (5%) using ImageJ software. Expression levels were quantified from gel images from pellicle and biofilm-related genes compared to its reference gene (*recA*). ⁣^∗^(*p* < 0.05) and ⁣^∗∗^(*p* < 0.01) are significantly different from the control as evaluated using *t*-test.

The semiquantitative analysis of *bcsA*, *fis*, *hrpL*, and *expI* gene expression was done to identify the effect of manuka honey on the expression of genes involved in biofilm and pellicle formation. The results suggested differences in expression among four of the genes studied (i.e., *bcsA* (*p* = 0.0453), *fis* (*p* = 0.0093), *hrpL* (*p* = 0.135), and *expI* (*p* = 0.0496)) without and with 5% manuka honey treatment (Figure 3(a)). Specifically, higher expression was observed in genes without manuka honey treatment than in genes with honey treatment (Figure 3(b)). The semiquantitative test results suggested that manuka honey reduces the expression of *bcsA*, *fis*, *hrpL*, and *expI*.

### 3.4. Quantitative Analysis of Gene Expression

**Figure 4:**
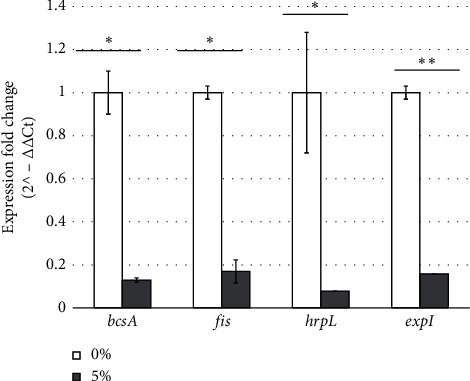
Alterations in the expression profiles of genes involved in the biofilm and pellicle formation of *Pectobacterium brasiliense* treated with 5% manuka honey as determined by RT-qPCR. All genes were downregulated, and different degrees of downregulation were observed. The expression of *bcsA*, *fis*, *hrpL*, and *expI* decreased by 7.07-fold, 5.71-fold, 13.11-fold, and 6.26-fold, respectively, after exposure to 5% (w/v) manuka honey. The fold change differences were calculated by dividing the expression fold change of 0% treatment by the expression fold change of 5% treatment for each biofilm and pellicle-related gene. ⁣^∗^(*p* < 0.05) and ⁣^∗∗^(*p* < 0.01) are significantly different from the control as evaluated using *t*-test.

We quantitatively analyzed the expression of the genes by using RT-qPCR to confirm the results of the semiquantitative test. The results showed a significant decrease in the expression of four genes of *P. brasiliense*, including *bcsA* (7.07-fold; *p* = 0.0198), *fis* (5.71-fold; *p* = 0.0446), *hrpL* (13.11-fold, *p* = 0.0163), and *expI* (6.26-fold; *p* = 0.0084), following treatment with 5% manuka honey (Figure 4). These results confirmed that manuka honey reduced the expression of genes involved in biofilm and pellicle formation.

## 4. Discussion

Biofilms and pellicles are formed by bacteria as a means of protection and self-defense against environmental factors. In this study, the observed biofilm in the SOBG medium is similar to that of the air–liquid interface (pellicle), which forms purple rings on the top of the PVC surface when staining with CV; however, the difference between treatment and control is well observed. Under the conditions tested, treatment with manuka honey at the lowest concentration tested (i.e., 5%) reduced biofilm formation, while treatment at the highest concentration tested (i.e., 75%) completely inhibited biofilm formation in *P. brasiliense*. The higher the concentration of manuka honey, the greater the extent of reduction of biofilm formation. This result may be attributed to various compounds acting together in manuka honey that can inhibit the formation of biofilms by pathogenic bacteria. Treatment with 5% manuka honey could also inhibit pellicle formation under static conditions. These findings might help to understand and identify the potential management of biofilm. In *Streptococcus pyogenes*, manuka honey can inhibit biofilm by inhibiting adherence, intracellular aggregation, and bacterial attachment to human tissue protein fibronectin [28]. Decreases in biofilm formation are influenced by methylglyoxal (MGO) and the sugar components of manuka honey; studies indicate that anti-biofilm activity is achieved not by a single inhibitor but by a combination of complex synergistic components [43, 44].

As described by Ava et al. [31], manuka honey could inhibit the growth of *P. brasiliense*. In this study, we described that biofilm formation was not only affected by cell number but also by the decreased expression level of biofilm-related genes. The decrease in biofilm and pellicle formation observed in this study is supported by decreases in the expression of *bcsA*, *fis*, *hrpL*, and *expI* at the semiquantitative and quantitative levels under the conditions tested. The *bcsA* gene encodes for bacterial cellulose synthase (*bcs*), which plays a role in pellicle and biofilm formation. The *bcsA* gene is encoded by the *bcs* operon, and *bcsA* transcription is controlled by *cytR* homolog; also, reducing *bcsA* expression is followed by reducing biofilm formation at the air–liquid interface [45]. Lv et al. [46] suggested that bacterial cellulose is involved in the formation of bacterial or biofilm communities. Besides BcsA, Fis is also closely related to *bcs* operons during biofilm/pellicle formation. The expression of the *bcs* operon is induced in the biofilm, directly regulated by Fis, and directly suppressed by interacting with the missing operator from the *bcs* operon in plant pathogenic *Pectobacterium* [47]. In *Dickeya zeae*, the *fis* deletion mutant produces fewer surface-attached biofilms compared with the wild-type variant [48].

The formation of biofilms in *P. brasiliense* in this study was influenced by QS, as indicated by the observed decrease in the expression of QS-related genes. The expression level of *expI* is positively correlated with biofilm. The *expI* functions to synthesize AHL, which is a signal released by bacteria for QS [45, 46]. Due to the high concentration of cells in biofilm, QS cell density-dependent regulation of gene expression plays a crucial role in physiological biofilm formation [49]. Since the cells in the biofilm aggregates are remarkably similar and are connected through a self-generated extracellular matrix, the biofilm represents an ecological environment related to QS [50, 51]. In addition, this study showed that pellicle formation also has a positive correlation with the expression of *hrpL*. Some studies reported that one of the factors that delay or reduce biofilm production at the air–liquid interface (pellicle) is the low expression of *hrpL* [52]. In plant pathogenic Pectobacteriaceae, such as *Pectobacterium*, *hrpL* encodes alternate sigma factor that functions as the main regulator genes in *hrp* (hypersensitive response and pathogenicity) cluster and also regulates the expression of type III secretion system (T3SS) [53, 54]. T3SS is required for the formation of bacterial aggregates at the air–liquid interface [55].

Air–liquid interface formation by plant pathogenic bacteria has been shown to be positively related to their virulence in plants [56, 57]. Biofilm-like structures of *P. brasiliense* in the xylem vessels of host plants may become important virulence factors for pathogenesis. Islamov et al. [58] reported that bacterial emboli or biofilm-like structures will be created when soft rot phytopathogenic bacteria colonize the xylem vessels of the host plant.

## 5. Conclusion

The treatment with 5% manuka honey could reduce the formation of biofilms and pellicles by *P. brasiliense*. This decrease is accompanied by reduction in the semiquantitative and quantitative gene expression of *bcsA*, *fis*, *hrpL*, and *expI*. In this study, there was no evidence that honey accelerated direct control of soft rot disease in *planta*; however, this study can be a basis for further research to understand the role of honey in suppressing biofilm and bacterial pathogenesis. Future studies on the direct application of honey to plant tissue using the bioactive compound of manuka honey in the prevention of bacterial soft rot might be required.

## Figures and Tables

**Table 1:** Primer sequences used for the RT-qPCR analysis of genes related to biofilm and pellicle formation in *Pectobacterium brasiliense.*

Gene	Accession	Function	Sequence (5′⟶ 3′)
*bcsA*	57242389	Cellulose synthase	F: AAGAAATGGTGCGGGGTTTG
			R: TCAACAACGCCCTGAAACAG

*fis*	57242014	DNA-binding protein	F: ACAACGCGTGAATTCTGACG
			R: TCCTGACCGTTCAATTGAGC

*hrpL*	AJ496800	Alternative sigma factor	F: AAGTCAGCGGTCCTTGAAAC
			R: TCCAACTGCAATGCGAGATC

*expI*	LC387225.1	Quorum-sensing signal generator, acyl-homoserine lactone	F: TGTCCCGGTAATCATGTTAGGG
			R: AATTGGGCCGTGCAATGTAC

*recA* ∗	57243346	Recombinase A	F: TGCGTTTATCGATGCTGAGC
			R: AGCGCGTTAATGCATCACAG

^∗^ *recA* was used as a reference gene.

## Data Availability

All data are provided in the article.
